# Tropical Forest Fragmentation Affects Floral Visitors but Not the Structure of Individual-Based Palm-Pollinator Networks

**DOI:** 10.1371/journal.pone.0121275

**Published:** 2015-03-31

**Authors:** Wesley Dáttilo, Armando Aguirre, Mauricio Quesada, Rodolfo Dirzo

**Affiliations:** 1 Instituto de Neuroetología, Universidad Veracruzana, Xalapa, Veracruz, Mexico; 2 Red de Interacciones Multitróficas, Instituto de Ecología, A.C., Xalapa, Veracruz, Mexico; 3 Escuela Nacional de Estudios Superiores, campus Morelia, Morelia, Michoacán, Mexico; 4 Centro de Investigaciones en Ecosistemas, Universidad Nacional Autónoma de México, Morelia, Michoacán, Mexico; 5 Department of Biology, Stanford University, Stanford, California, United States of America; Arizona State University, UNITED STATES

## Abstract

Despite increasing knowledge about the effects of habitat loss on pollinators in natural landscapes, information is very limited regarding the underlying mechanisms of forest fragmentation affecting plant-pollinator interactions in such landscapes. Here, we used a network approach to describe the effects of forest fragmentation on the patterns of interactions involving the understory dominant palm *Astrocaryum mexicanum* (Arecaceae) and its floral visitors (including both effective and non-effective pollinators) at the individual level in a Mexican tropical rainforest landscape. Specifically, we asked: (i) Does fragment size affect the structure of individual-based plant-pollinator networks? (ii) Does the core of highly interacting visitor species change along the fragmentation size gradient? (iii) Does forest fragment size influence the abundance of effective pollinators of *A*. *mexicanum*? We found that fragment size did not affect the topological structure of the individual-based palm-pollinator network. Furthermore, while the composition of peripheral non-effective pollinators changed depending on fragment size, effective core generalist species of pollinators remained stable. We also observed that both abundance and variance of effective pollinators of male and female flowers of *A*. *mexicanum* increased with forest fragment size. These findings indicate that the presence of effective pollinators in the core of all forest fragments could keep the network structure stable along the gradient of forest fragmentation. In addition, pollination of *A*. *mexicanum* could be more effective in larger fragments, since the greater abundance of pollinators in these fragments may increase the amount of pollen and diversity of pollen donors between flowers of individual plants. Given the prevalence of fragmentation in tropical ecosystems, our results indicate that the current patterns of land use will have consequences on the underlying mechanisms of pollination in remnant forests.

## Introduction

Deforestation is the major cause of fragmentation and spatial isolation of populations in the tropics [1], with potential to increase species or population extinction rates [1,2]. Despite the importance of ecological interactions in the structure and stability of biological communities over time and space [3], most studies have focused only on populations and species loss, but studies on the consequences of fragmentation on ecological processes are gaining prominence in the literature [4, 5, 6, 7]. Indeed, recent research indicates that species interactions are increasingly at risk of local and global extinction as a consequence of changes in land use [7, 8, 9].

Fragmentation is one of the major threats to plant-pollinator interactions in the tropics [7, 10, 11]. This occurs mainly because the loss of habitat decreases pollinator activity, pollen deposition, and outcrossing levels, which directly influences the reproductive success of plants remaining in isolated fragments [7, 12, 13]. Recent studies have also suggested that there is a global pollination crisis due to the decline in both abundance and richness of pollinators [14, 15]. Under a unified framework of species interactions, studies have focused on the structural properties of plant-pollinator networks at the community level in different systems and habitats throughout the Earth [16, 17, 18, 19], including the effects of habitat loss on these networks [20, 21, 22]. The topological structure of these ecological networks can be described through patterns of species interactions such as nestedness, modularity, specialization and diversity of interactions (reviewed by [23]). Nestedness describes the degree to which specialists interact with a subset of the species interacting with generalists. Modularity describes the presence of semi-independent compartments of highly interacting species. Specialization refers to the overall level of dependence of all interacting species in a network. Diversity of interactions describes the magnitude of the interactions between plants and animals. Despite the increase in knowledge on plant–pollinator networks at the community level, few studies have evaluated how these mutualistic relationships vary within biological populations, but see [24].

We know that within a given population of a plant species, there is variation in the frequency of flower visitation within and among individual plants [25, 26, 27]. One of the main possible factors explaining such intrapopulation variation is based on differences in the quality of reward, and therefore it would be expected that individuals with better rewards would be most visited, ensuring cross-pollination. However, plants with longer flowering time could also be the most visited. Consequently, such inter-individual variation can directly affect the fitness of plants, since it is expected that the most visited individuals will have greater reproductive success (seed and fruit set) [26, 27]. Variation in pollinator visitation frequency to flowers can generate complex networks among individuals of the same plant species. Similar studies have been conducted involving individual-based networks in other interaction systems using an intrapopulation approach, such as sea otters-prey, didelphid marsupials-arthropods, snails and fruits, monkeys-fruiting plants, frogs, ant-plants, and lizards-diets [28, 29, 30, 31, 32, 33]. However, to our knowledge only two studies have evaluated the networks of plant-pollinator interactions among individuals within populations [26, 27].

In tropical rainforests, palms are an important component of the understory [34, 35, 36]. It is estimated that there are between 2400 [37] and 2700 palm species [38] in tropical and subtropical regions around the world, yet little is known about pollination interactions and reproduction of these plants in fragmented landscapes [39]. Since each palm tree can be visited by a rich contingent of different insect groups [40, 41], we examined if these plant-insect interactions could be a good model to study individual-based pollination networks. To our knowledge, this is the first study that directly evaluates intrapopulation variations in pollinator activity of palms in fragmented landscapes. Here, we used a network approach to describe the effects of forest fragmentation on the patterns of interactions involving the understory dominant palm *Astrocaryum mexicanum* (Arecaceae) and its floral visitors. For this, we collected effective (Coleoptera: Nitidulidae) and non-effective pollinators on male- and female-phase inflorescences in populations of *A*. *mexicanum* (Arecaceae) in different tropical rainforest fragments of Los Tuxtlas, in southeast Mexico. We hypothesized that the size of the forest fragment could affect the abundance of effective and non-effective pollinators of *A*. *mexicanum*, and consequently, influence the pattern of interactions within the networks. We then addressed the following questions: (i) Does fragment size affect the structure of individual-based plant-pollinator networks? (ii) Does the core of highly interacting visitor species change along the fragmentation size gradient? (iii) Does forest fragment size influence the abundance of effective pollinators of *A*. *mexicanum*?

## Materials and Methods

### Ethics statement

All research was conducted in accordance with national and international guidelines, and conforms to the legal requirements of the Mexican government. Los Tuxtlas Biological Station-UNAM granted permission to conduct the study in all selected fragments.

### Study site and fragments

This study was conducted at the Los Tuxtlas Biological Station, a field station of the National University of Mexico (UNAM) (18°34′–18°36′ N, 95°04′–95°09′ W) located in the State of Veracruz, Mexico. The area has a complex topography with elevation ranging from sea level to 1650 m above sea level within a short distance [42]. The predominant land use in the area is conversion of forest to cattle grasslands, and rates of deforestation in the decades of the 60’s-late 80’s were ca. 4% per year [43]. Current deforestation rates have decreased because forest remnants are restricted to the most inaccessible areas of the region [44]. Floristic diversity in the area includes a total of 950 known vascular plants [45], which largely consist of plants of Neotropical origin, but higher elevation sites include combinations of Neotropical and Nearctic taxa [42]. Palms are a very important component of the tropical rainforest of Los Tuxtlas [45] with 13 species present [46], but only a few of them, such as *Astrocaryum mexicanum*, *Chamaedorea pinnatifrons*, *Ch*. *alternas*, *Ch*. *Concolor*, *Ch*. *tepejilote* and *Bactris mexicana* attain high population densities [45, 47].

In this study, we sampled six forest fragments encompassing a size-range of 2, 4, 19.4, 34.6, 114.6 and 700 ha, the latter representing the reference site, as it extends along the entire elevational range, up to the maximum elevation in the area. Isolated fragments were selected based on the following characteristics: i) they bore the same vegetation type, tropical rainforest, and therefore were located within a restricted elevation (0–150 m a.s.l.); ii) similar age (<50 years of separation); and iii) all plants were located within a plot of 600 m^2^ established at the center of each forest fragment and continuous forest to avoid edge effects [39, 48].

### Species studied and data collection

**Fig 1:**
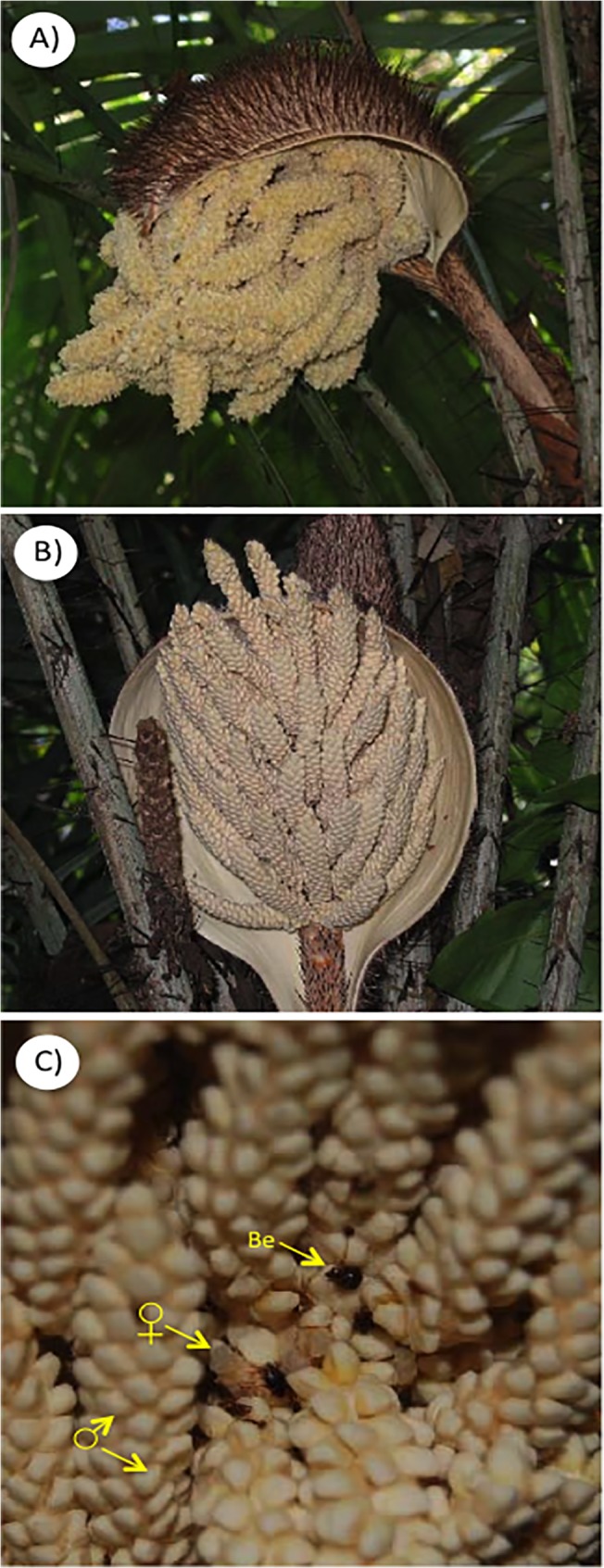
Male phase (A) of inflorescence of *Astrocaryum mexicanum* showing open male flowers releasing large quantities of pollen, and female phase (B) showing numerous rachillae with male flowers closed. Female flowers (C) located inside an inflorescence surrounded by rachillae, and one of the main pollinators, the beetle *Coleopterus aberrans* (Be).

*Astrocaryum mexicanum* is the only species of the genus in Mexico [33], reaching densities of up to 1,000 individuals (> 1 m tall) per hectare at Los Tuxtlas [44, 48]. Plants are monoecious (dichogamous), with up to five inflorescences per plant in both sexual phases of the inflorescences [49] (Fig. 1A and 1B). The effective pollinators are small nitidulid beetles (*Mystrops* sp., *M*. *mexicanus*, *Eumystrops centralis* and *Coleopterus aberrans*) (Fig. 1C), however, many other animal species can visit the flowers of *A*. *mexicanum* [47, 49]. Previous studies have shown that *A*. *mexicanum* supports a great diversity of floral visitors (10 arthropod orders, 60 species), and that habitat fragmentation negatively affected the abundance of the main pollinators, but did not have an effect on the plant´s reproductive success [39]. The abundance of potential pollinators (Coleoptera: Nitidulidae) is striking, ranging from 1270 individuals/plant in small fragments (2ha) to 9,893 individuals/plant in the largest fragment (700 ha) [39]. Within each fragment we established a permanent plot of 600 m2 to collect the inflorescences, and we tagged all reproductive plants of *A*. *mexicanum*. From these we selected a group of reproductive plants of similar height (2 m) and age (~40–50 years) to sample flower visitors within each fragment. Visitors to the inflorescences were collected during the peak flowering season (March-May) of 1999. Within each fragment we collected the inflorescences of 10 and 5 randomly selected palms in the female and male sexual phases, respectively, when the activity of flower visitors is more intense [39]. Inflorescences were collected between 0700 and 0800 AM, the time at which we observed the most intense insect activity, according with observations by Búrquez et al. [50].

### Data analysis

Initially, we used a paired t-test (paired per fragment) to evaluate the differences in the number of floral visitor species and the abundance of effective pollinators between male and female inflorescences. To evaluate the effects of forest fragmentation on the patterns of interactions involving *Astrocaryum mexicanum* and their floral visitors, we used a network approach and defined each of our six fragments as an adjacency matrix *A* (or interaction network), where *a*_*ij*_ = number of interactions from an individual plant *j* by the floral visitor species *i*, and zero for absence of interactions [51]. Therefore, we built six different interaction networks according to size of the forest fragment.

To evaluate the specialization of our visitation networks based on quantitative data (i.e., interaction frequencies), we used the specialization index (*H*_*2*_’) [52]. In this index, extreme generalization is *H*_*2*_’ = 0 and extreme specialization is *H*_*2*_’ = 1. By incorporating frequency data, this specialization index is extremely robust to changes in sampling intensity and the number of interacting species compared to most other specialization indices (see more details in: [52,53]. We also calculated the diversity of interactions (*ID*) through a widely used index for analyses of interaction networks [54, 55, 56, 57].

We also performed a second approach that involves the search for non-random patterns of plant-floral visitor interactions. We estimated nestedness for each network using the NODF-metric [58] in ANINHADO [59]. Values of this metric range from 0 (non-nested) to 100 (perfectly nested). In addition, we tested whether within each network there were groups of floral visitors strongly associated with a particular set of individual plants. For this we used the modularity index (*M*) based on Simulated Annealing (*SA*) (range 0–1) [60, 61] using the software MODULAR [62]. This index ranges from 0 = no subgroups, to 1 = totally separated subgroups [16]. Although this index *M* is used for unipartite networks, our null models control any potential effects of bipartite structure on modularity (interactions only occur between floral visitors and plants) [29, 63]. We tested the significance of NODF and M for each network through 1000 simulated networks generated by the Null Model II (CE) to assess whether the observed values in the empirical networks were higher than would be expected for null distributions of these values [64]. In this null model, the probability of an interaction occurring is proportional to the number of interactions of both floral visitors and plant individuals [51]. We used these network descriptors and null model because they provide a way to characterize the organization of these networks in a way that allows direct comparison with previous work on individual-based networks. We defined floral visitor species as core (*i*.*e*., those with the highest number of interactions) or peripheral (*i*.*e*., those with the fewest number of interactions) components of the network according to [65].

To explore the effects of forest fragment size on plant-floral visitor networks, we used simple linear regression with the log_10_-transformed fragment size as a predictor variable for the following network descriptors: richness of floral visitors, network specialization, interaction diversity, nestedness and modularity. We also used simple linear regression to evaluate the effects of forest fragment size on the abundance of effective pollinators on male and female inflorescences.

We tested for differences in peripheral and generalist core species among fragments through a permutation test (10,000 permutations) using Analysis of Similarities (ANOSIM) based on Bray-Curtis’s dissimilarity index (quantitative data). All data analyses were performed using R–software version 3.1.2 [66]. All data are included within the manuscript.

## Results

**Fig 2:**
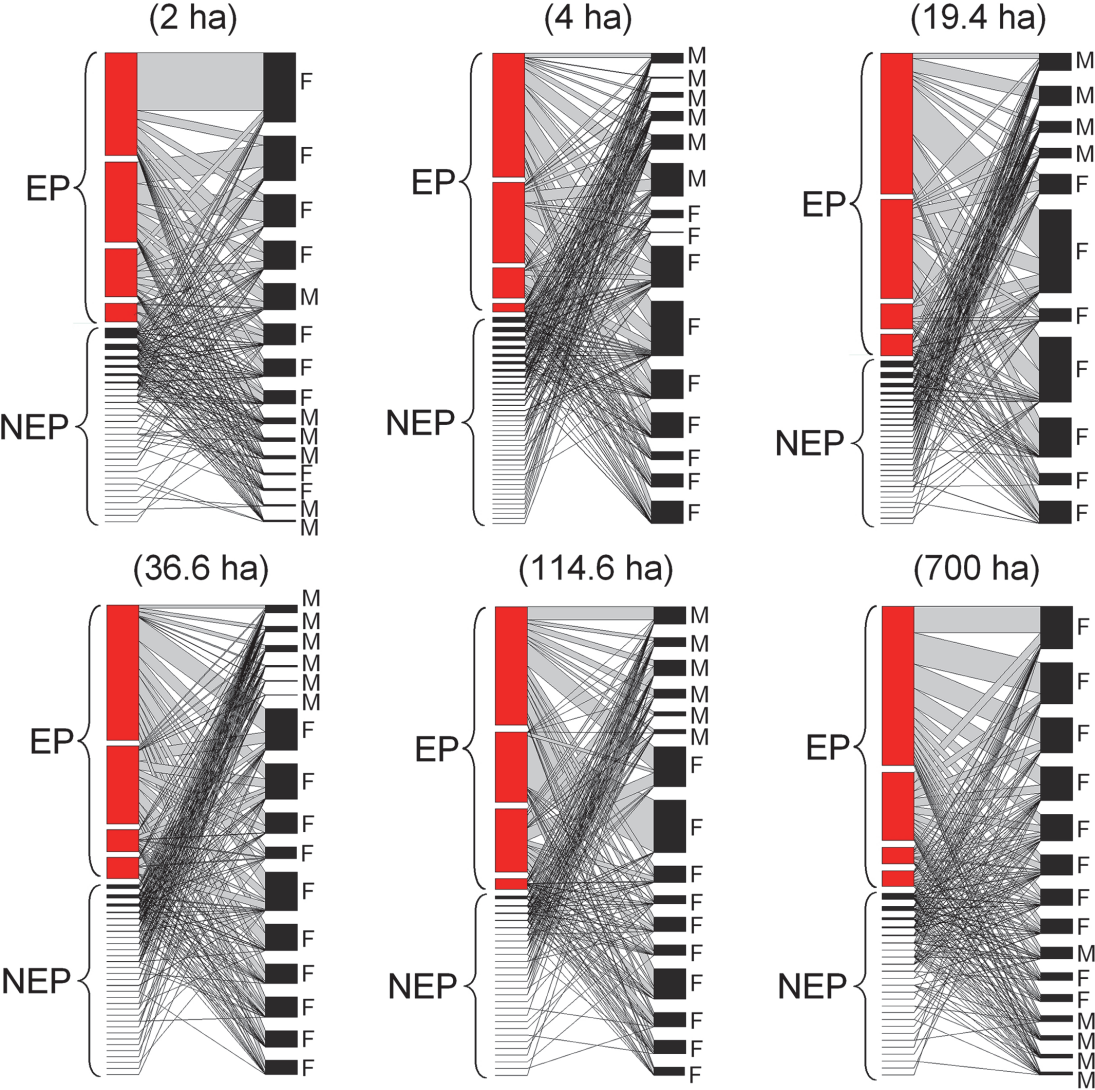
Individual-based networks involving individuals of *Astrocaryum mexicanum* (Arecaceae) and their effective (EP) and non-effective (NEP) pollinators in six tropical rainforest fragments (2, 4, 19.4, 34.6, 114.6 and 700 ha respectively) of Los Tuxtlas, southeast Mexico. The right nodes represent different individuals of *A*. *mexicanum* considering both (M) male- and (F) female-phase inflorescence. The left nodes correspond to species of floral visitors that interact with plant individuals. Lines indicate interactions between the two trophic levels. Networks were ordered by both number of links and interaction frequencies. Rectangle height is proportional to the number of interactions recorded per species. Different line lengths indicate the frequency of interactions.

Across all fragments, we collected a total of 228,772 arthropods (10 orders, 60 species or morphospecies) visiting inflorescences of *A*. *mexicanum* individuals (considering both male and female phases) (Fig. 2). Coleoptera was the predominant group (> 50% of the species) including the four main pollinators: *Eumystrops centralis*, *Mystrops mexicanus*, *Coleopterus aberrans*, and *Mystrops* sp. (Coleoptera), followed by Hymenoptera (20%), while the remaining (30%) was distributed among the other eight orders. The average number of floral visitors per individual of *A*. *mexicanum* was 34.83 species (range from: 30 to 42 species). Of the 60 floral visitor species collected, just one to eight species were present in the central core of highly generalist species: *E*. *centralis* (Coleoptera), *M*. *mexicanus* (Coleoptera), *C*. *aberrans* (Coleoptera), *Mystrops* sp. (Coleoptera), Unidentified sp1 (Diptera), Unidentified sp1 (Coleoptera), Unidentified sp2 (Coleoptera). Male inflorescences had higher numbers of floral visitor species (Mean ± SD: 18.52 ± 1.93 species) when compared with female inflorescences (12.49 ± 0.79 species) (*t* = −3.8; *df* = 5; *p* = 0.01). However, the abundance of pollinators per female inflorescence was higher (530.63 ± 813.04) compared to male inflorescences (425.31 ± 530.63) (*t* = −2.755; *df* = 5; *p* = 0.04).

**Fig 3:**
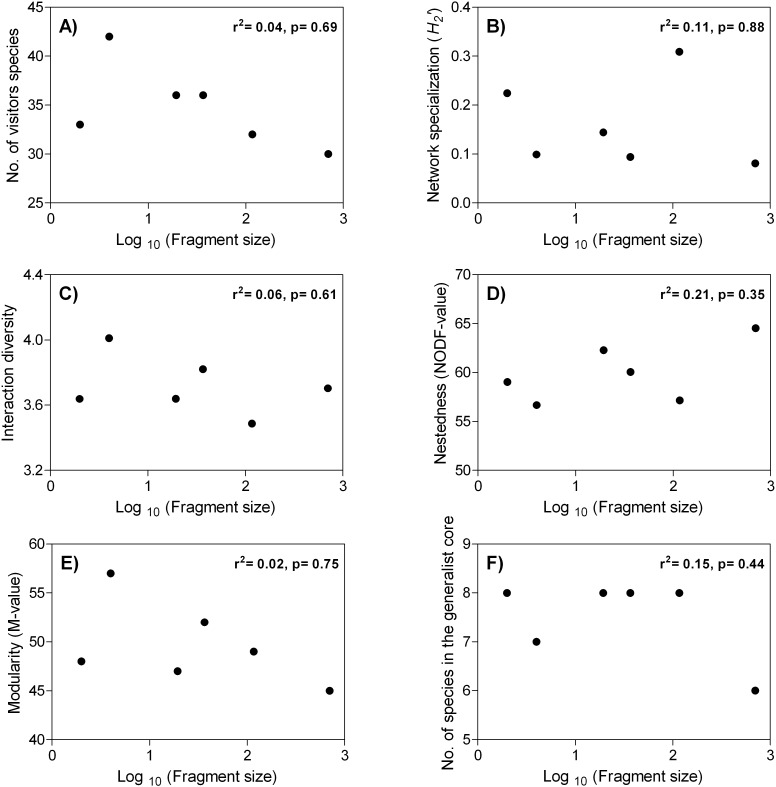
Relationship between: number of floral visitor species (A); network specialization (*H*_*2*_’) (B); interaction diversity (C); nestedness (NODF-metric) (D); modularity (M-metric) (E); number of visitor species found in the central core of highly generalist species (F), and the log_10_-transformed fragment size of six fragments in Los Tuxtlas, southeast Mexico. Regression correlation coefficient (*r*) and significance (*p*) computed using simple linear regressions are also shown.

**Fig 4:**
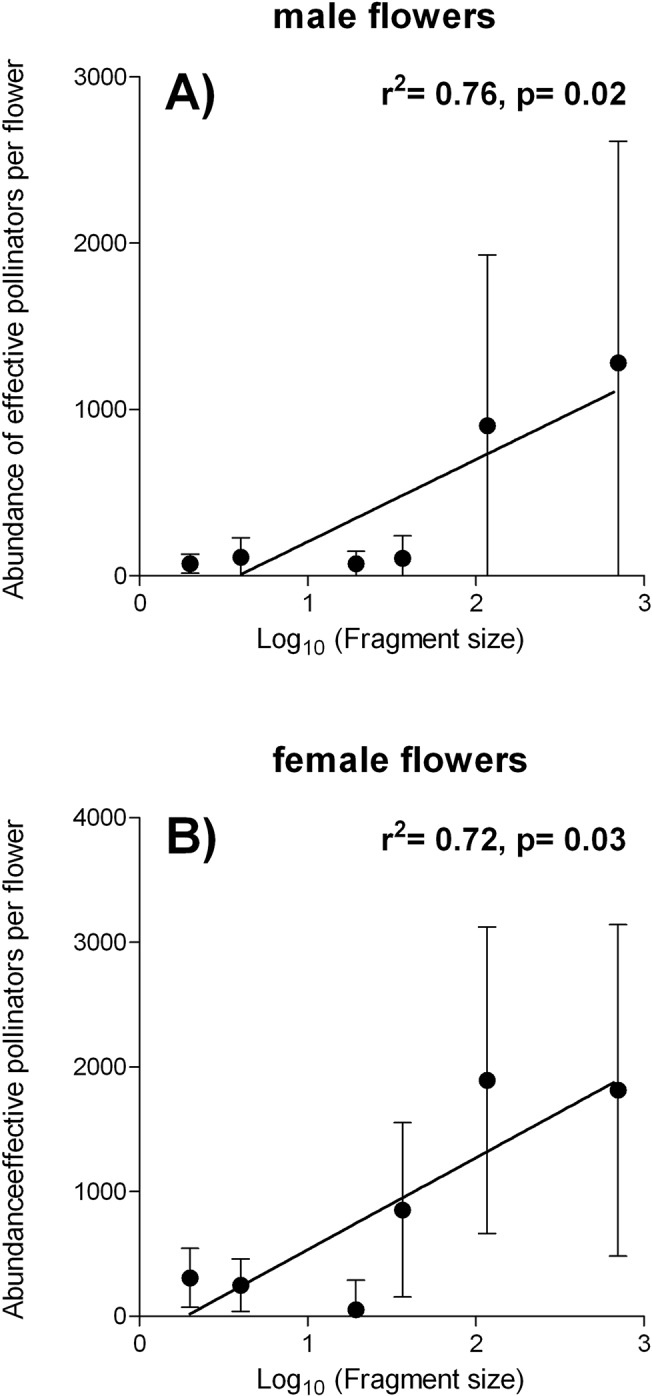
Forest fragment size effects (log_10_-transformed) on the abundance (± SD) of effective pollinators per (A) male and (B) female inflorescence of *Astrocaryum mexicanum* (Arecaceae) in Los Tuxtlas, southeast Mexico.

When we evaluated the effect of forest fragmentation on the interaction networks, we found that the number of visitor species within each network did not change regardless of the fragment size (Mean ± SD: 34.83 ± 4.21 species. *r* ^*2*^ = 0.042, *f* = 0.176, *df* = 1, *p* = 0.697) (Fig. 3A). In general, we found a low level of network specialization (*H*_*2*_’ = 0.16 ± 0.11) and no trend in variation in the specialization values across fragment sizes (*r* ^*2*^ = 0.114, *f* = 0.023, *df* = 1, *p* = 0.886) (Fig. 3B.) The mean interaction diversity index in our networks was 3.71 (± 0.17) and also remained stable over the fragments studied (*r* ^*2*^ = 0.069, *f* = 0.297, *df* = 1, *p* = 0.615) (Fig. 3C). We evaluated non-random patterns of species interaction within each network and found that the six networks exhibited a significantly nested network topology (all *p*-values < 0.01), with *NODF*-values ranging from 56.68 to 64.54. In this case, we observed that plant individuals with higher number of interactions were visited by both effective pollinators and non-pollinators while individuals with few interactions were only visited by pollinators, generating the cohesive subgroups expected in nested networks. On the other hand, no network was significantly modular when compared with the neutral patterns of interactions (null models) (Modularity values ranged from 0.151 to 0.208.) Both nestedness and modularity values did not vary according to the size of the fragments (Nestedness: *r* ^*2*^ = 0.217, *f* = 1.109, *df* = 1, *p* = 0.352. Modularity: *r* ^*2*^ = 0.027, *f* = 0.110, *df* = 1, *p* = 0.757) (Fig. 3D and 3E). In addition, we observed that abundance and the standard deviation of effective pollinators per male and female inflorescence of *A*. *mexicanum* significantly increased with forest fragment size (male: *r* ^*2*^ = 0.76, p = 0.02; female: *r* ^*2*^ = 0.72, *p* = 0.03) (Fig. 4).

*Regression* correlation *coefficient* (*r*) and significance (*p)* computed using simple linear regressions are also shown.

We observed that the richness of flower visitor species changed within each network (from six to eight species). However, richness of flower visitor species was not related to the size of fragments (*r* ^*2*^ = 0.154, *f* = 0.729, *df* = 6, *p* = 0.441) (Fig. 3F). Finally, although the species composition of floral visitors found in the network periphery changed between fragments (ANOSIM: *r* = 0.208, *p*<0.001), the central core of highly generalist visitor species remained stable (ANOSIM: *r* = 0.021, *p* = 0.123).

## Discussion

In this study, standardizing the sampling effort based on male- and female-phase inflorescences, we empirically showed for the first time that individual-based palm-pollinator networks are highly nested and non-modular and the sizes of the fragments studied did not affect the topological structure of these networks. Moreover, although the network periphery (*i*.*e*, those with fewer interactions) of non-effective pollinators changed across fragments, core generalist species of effective pollinators remained stable over the spatial scale studied. We also observed that both abundance and variance of effective pollinators per male and female inflorescence of *A*. *mexicanum* increased with forest fragment size. Theses findings indicate that the presence of effective pollinators in the core of all forest fragments could keep the structure of the networks stable along the gradient of forest fragmentation. However, pollination of *A*. *mexicanum* could be more effective in larger fragments, since the greater abundance of pollinators in these fragments could increase the amount of pollen and diversity of pollen donors transferred between flowers.

In individuals of *A*. *mexicanum*, anthesis of male and female inflorescences occurs in local asynchrony [50]. Interestingly, female inflorescences open their flowers at 05:00 AM and increase the temperature inside the inflorescence up to 13.5°C (reaching a maximum of 28°C) [50]. Based on these two mechanisms (dichogamy and thermogenesis), we should expect female inflorescences to have a greater richness of floral visitors. However, we observed that male inflorescences have higher richness of visitors per individual plant in all fragments studied. Thermogenesis is an interesting mechanism found in some angiosperm families including Arecaceae [67, 68]. The ecological implications of thermogenesis on pollination in palms are diverse and unknown in most cases, but it has been associated with 1) pollen growth tube, 2) stimulation to pollinators to leave the inflorescences, 3) diffusion of volatiles, and 4) an increase of temperature as a growth-promoting factor to eggs and larvae [68]. According to Búrquez et al. [50], most visitors of *A*. *mexicanum* concentrate their activities in two types of rewards: feeding on the petals of male inflorescences and pollen consumption. This is because female inflorescences have lignified cells (sclerenchyma) and acicular crystals (raphides) that may protect their structures against herbivory [50]. Protective attributes have been reported in inflorescences in different species of the dioecious palm *Chamaedorea* sp., where certain morphological features in the flowers including sclerified tissue, silica bodies and raphide-containing ideoblasts are represented differentially in the sexual phases of palms [68, 69]. The greatest protection against herbivory in female inflorescences is possibly associated with protection of the fruits that will develop later. Therefore, the largest richness of visitors on male inflorescences is associated with herbivorous species, which consume flowers and pollen of *A*. *mexicanum* mainly due to high nutritional content in terms of proteins, lipids and carbohydrates [70, 71]. In addition, other species are attracted to male flowers to prey on some arthropods visiting these flowers [50].

Based on previous studies, we know that mutualistic networks are significantly nested when evaluated at the community level (e.g., seed dispersal, pollination, and protective networks) [51, 72, 73]. However, the nested pattern has only recently been reported in other types of ecological networks at the intrapopulation level [28, 29, 31, 74]. Here we showed that nestedness is also a non-random pattern of individual-based palm-floral visitor networks. This indicates that the floral visitors of *A*. *mexicanum* did not forage opportunistically, since the less visited individuals (visited only by pollinators) are a subset of the more visited individuals (visited by both effective and non-effective pollinators). We hypothesize that variation between individual plants (e.g. floral rewards) associated with floral resources might be the main traits that generated nested patterns [27]. Moreover, we observed that within the populations studied, there are no groups of pollinator species visiting more frequently a particular group of individual plants and generating highly modular networks. This can occur because all floral visitors of a plant species must have the same ability to visit all the individuals of such species. Therefore, it is expected that pollination networks can be highly modular only when different plant species are evaluated, in which some plant species are adapted to particular groups of pollinators (i.e. the classical definition of pollination syndromes) [16, 75], and due to specialization to floral characteristics by the effective pollinators [76]. Additionally, it is possible that the sequential flowering of this palm’s phenology can also generate modular networks, once the first plants to flower could not be visited by the same visitors than more delayed flowering plants [77], which generate compartments with many intragroup links and few intergroup links.

We know that the few species found in the generalist core contribute considerably more to the nested pattern and to the stability of the network compared to peripheral species [23]. In this study, although the peripheral composition of floral visitors in the networks changed across fragments, the core of generalist species remained stable over all fragments studied. Moreover, these species of floral visitors found in the generalist core are certainly the effective pollinators of *A*. *mexicanum*, such as *E*. *centralis*, *M*. *mexicanus*, *C*. *aberrans*, and *Mystrops* sp. On the other hand, we did not find any effect of the fragment size on the structure of individual-based plant-pollinator networks, which does not corroborate our initial prediction. Therefore, it is possible that the effective pollinators are maintaining the structure of these networks stable along the fragmentation gradient, and local extinction of such species may lead to loss of populations of *A*. *mexicanum* and the collapse of the networks within these fragments.

In this study we showed that individual-based palm-floral visitor networks may be more stable to forest fragmentation when compared with plant-pollinator networks studied at the community level, e.g., [22]. In addition, we observed that the magnitude and variance of effective pollinators of male and female flowers of *A*. *mexicanum* increased with forest fragment size. In this case, small fragments where individuals are less visited by effective pollinators could exacerbate the amount of pollen and diversity of pollen donors transferred between flowers (less variance in the abundance of pollinators per flower). Also the density of reproductive individuals can lead to an increase of potential donors, and have a positive impact on pollination success, as showed for the Neotropical palm *Geonoma epetiolata* [78]. These fragmentation-related scenarios could have strong consequences on the maintenance of plant species if we consider breeding systems of plants, as well as size and mobility capacity of pollinators, with consequences on some attributes of performance of seedlings in forest remnants [79]. In sum, we showed that beyond the structure of interaction networks, intrinsic and biological factors of plant-pollinator interactions within such networks are crucial to our knowledge of the conservation and maintenance of pollination networks in tropical forests. Moreover, given the prevalence of fragmentation in tropical ecosystems, our results indicate that the current patterns of land use will have consequences on the underlying mechanisms of pollination in remnant forests.
